# Epidemiological status of dermatophytosis in Guilan, north of Iran

**DOI:** 10.29252/cmm.3.1.20

**Published:** 2017-03

**Authors:** AA Fallahi, A Rezaei-Matehkolaei, S Rezaei

**Affiliations:** 1 Department of Microbiology and Parasitology and Immunology, School of Medicine, Guilan University of Medical Sciences, Rasht, Iran; 2 Infectious and Tropical Diseases Research Center, Health Research Institute, Ahvaz Jundishapur University of Medical Sciences, Ahvaz, Iran; 3 Division of Molecular Biology, Department of Medical Mycology and Parasitology, School of Public Health, Tehran University of Medical Sciences, Tehran, Iran

**Keywords:** Dermatophyte, Epidemiology, Guilan, Tinea

## Abstract

**Background and Purpose:**

The epidemiological features of dermatophytoses have been characterized in many geographical locations of Iran, but not in Guilan, North of Iran. This study was carried out to determine the distribution pattern of dermatophytoses and their relevant agents in Guilan, North of Iran, over a period of one year, from April 2010 to April 2011.

**Materials and Methods:**

The clinical samples of skin, hair, and nail from 889 outpatients (317 men vs. 572 women) were used for direct microscopy and culture. All the culture-positive samples were then subjected to amplification of the internal transcribed spacer (ITS) of the nuclear rDNA followed by a restriction fragment length polymorphism (RFLP) assay to verify the causative agents.

**Results:**

The infection was confirmed in 90 (44.3%) males and 113 (55.7%) females. The most common type of dermatophytoses was tinea cruris (42.9%), followed by tinea pedis (20.2%), tinea corporis (11.3%), tinea unguium (7.4%), tinea faciei (6.9%), tinea manuum (6.4%), and tinea capitis (4.9%). ITS-RFLP based of the identification of isolates, showed that the infections were significantly associated with anthropophilic species, of *Trichophyton rubrum* (41.9%), *Epidermophyton floccosum* (19.7%), *T. tonsurans* (5.4%), and *T.* *violaceum* (2%). Other causative agents were *T. interdigitale* (22.6%), *Microsporum canis* (4.9%), *T. verrucosum* (2.5%), and *M. gypseum* (1%).

**Conclusion:**

The higher prevalence of *T. rubrum*, as the agent of dermatophytoses, than other species has never been reported from Iran and is of public health concern because of the chronic nature of infections with anthropophilic species. To thoroughly investigate the epidemiological trend of dermatophytoses in Iran, further periodical and molecular-based studies are necessary.

## Introduction

Ringworm infection or tinea (medically termed as dermatophytosis) is a spectrum of skin mycoses due to dermatophytes, a group of closely related fungi belonging to the *Trichophyton*, *Microsporum*, and *Epidermophyton* genera. They have the ability to utilize and invade the keratin, major structural protein of the skin, nails, and hair, and cause a variety of cutaneous infections that involve both humans and animals [1-3]. It is well known that the distribution patterns of dermato-phytoses and the relevant agents can no longer be restricted within a certain geographical region due to the development of tourism and migration activities, change in cultural habits (lifestyle), and socioeconomic and climate conditions. These issues have therapeutic and epidemiological significance; thus, to better understand the epidemiological trends of these infections, regular and successive updating of our knowledge in terms of the geographical patterns of dermatophytoses and pathogenic dermatophytes is mandatory [3-7]. In this regard, the recent advancements in molecular biology have led to the development of efficient, fast, and reliable DNA-based tools for the identification and epidemiological study of dermatophytes [8-11].

The epidemiological features of dermatophytosis, the most common superficial mycosis in Iran, were described in many parts of the country (i.e., Tehran, Ahvaz, Isfahan, Hamadan, Qazvin, and Shiraz) [12-17]; however, limited data is available for the epidemiological status of dermatophytosis in Guilan, Northern Iran [18]. 

Guilan is a province located on the southern coast of the Caspian Sea that geographically comprises a large coastal margin and has a humid subtropical climate and the highest rate of rainfall in Iran. Due to the marshy nature of the plains, the humidity rate is very high and reaches over 90% in summer. In this survey, we aimed to delineate the distribution profile of dermatophytosis and its corresponding agents in this province using a pre-described PCR-RFLP procedure on rDNA internal transcribed spacer (ITS) regions [12].

## Materials and Methods

***Isolates***

Over a one-year period, from April 2010 to April 2011, clinical specimens were obtained from outpatients suspected of dermatophytosis and referred to the Medical Mycology Laboratory at Guilan University of Medical Sciences, Rasht, Iran. All the individuals were asked to complete a form containing items on demographic and clinical information. The pathological samples (skin, hair, and nail) were obtained from each patient by scrapping and mounted in 20% potassium hydroxide (KOH) solution. Then, the samples were examined microscopically for verification of infection. Another portion of the sample was inoculated onto *Mycosel Agar* (BD Diagnostics, USA) plate and incubated at 28°C up to four weeks. The plates containing isolates that were identified as dermatophytes in the preliminary screening by gross and microscopic assessments were subjected to additional molecular analysis.

***Molecular identification***

The total genomic DNA was extracted from each isolate according to a previously described method [11]. In brief, a small piece of each fresh colony was ground by Micro Multi Mixer (*IEDA Co.* Ltd., Japan) in the presence of lysis buffer (200 mM Tris-HCl [pH: 7.5], 25 mM EDTA, 0.5% w/v SDS, and 250 mM NaCl). The homogenate was mixed with 300 µl of phenol-chloroform (25:24), then vortexed shortly and centrifuged at 10,000 × *g* for 10 min. The supernatant was mixed with an equal volume of chloroform and centrifuged again. The DNA pellet was precipitated by adding an equal volume of cold 2-propanol and 1/10 *volume of* 3 *M* sodium acetate at -20°C for 10 min, rinsed with 70% ethanol, air-dried, and dissolved in 50 μl of ultrapure water. The *rDNA* locus coding for the *5.8S* *rRNA* and *the flanking* internal transcribed spacers (ITS1-ITS2) were amplified using the universal fungal primers, ITS1 and ITS4 [19]. Amplification was carried out in a reaction mixture containing 5 µl of 10x PCR buffer, 1 µl of each forward and reverse primer (15 pM), 2.5 mM of dNTPs, 1 µl of genomic DNA, and enough sterile water to a final volume of 50 µl.

The amplification parameters were adjusted for: initial denaturation step at 94°C for 6 min, 35 cycles of 94°C for 30 s, 58°C for 30 s, and 72°C for 1 min, followed by an ultimate extension at 72°C for 10 min. A 10-μl aliquot of each amplicon was then digested with the restriction enzyme *Mva*I in a 30-μl reaction mixture according to the *manufacturer's instructions (*Thermo Fisher Scientific, USA). To confirm the success of molecular experiments, 15 µl of each PCR and RFLP product was respectively electrophoresed on 1% and 2% agarose gel in Tris-acetate EDTA buffer, stained with ethidium bromide (0.5 µg/ml), visualized under UV light, and photographed. For the identi-fication of all the isolates, the restriction pattern of each colony was compared with those specific profiles illustrated in the previous report on dermatophyte species [18].

## Results

The 889 samples obtained from the patients with clinical signs were suggestive of dermatophytosis, including 572 women and 317 men. Of these, 203 (22.8%) specimens were positive for infection (i.e., 90 (44.3%) males and 113 (55.7%) females; Table 1). The majority of the surveyed individuals engaged in agricultural activities and were predominantly from rural areas. Regarding the age distribution, the patients were within the age range of 1-82 years, and the 20-30 age group was the most frequent group (Table 2). The infections occurred slightly more frequently in summer. With regards to the distribution of clinical forms, involvement of the groins (tinea cruris) was the most common type (42.9%), followed by tinea pedis (20.2%), tinea corporis (11.3%), tinea unguium (7.4%), tinea faciei (6.9%), tinea manuum (6.4%), and tinea capitis (4.9%). The overall prevalence of dermatophytosis was higher in females than males, except for tinea unguium, which was more frequent in men (4 vs. 11). Tinea faciei and tinea capitis were equally distributed in *both* *genders (Table 2). *

In molecular analysis, rDNA ITS-RFLP led to the identification of anthropohilic species, namely *Trichophyton rubrum* (41.9%), *Epidermophyton floccosum* (19.7%), *T. tonsurans* (5.4%), and* T.* *violaceum* (2 %), as the main causes of infection.

**Table 1:** Frequency of dermatophyte isolates with regard to the type of tinea and patients' gender in Guilan

**Clinical form Species**	***T. cruris***	***T. pedis***	***T. corporis***	***T. unguium***	***T. faciei***	***T. manuum***	***T. capitis***	**Total (%)**	**(M/F)** ^*^
*T. rubrum*	44	16	11	5	3	6	0	85 (41.9)	(39/46)
*T. interdigitale*	11	20	3	9	2	1	0	46 (22.6)	(18/28)
*E. floccosum*	30	3	5	0	0	2	0	40 (19.7)	(17/23)
*T. tonsurans*	0	0	2	0	6	0	3	11 (5.4)	(10/1)
*M. canis*	1	1	0	1	2	1	4	10 (4.9)	(2/8)
*T. verrucosum*	0	0	1	0	0	1	3	5 (2.5)	(3/2)
*T. violaceum*	1	0	1	0	0	2	0	4 (2)	(1/3)
*M. gypseum*	0	1	0	0	1	0	0	2 (1)	(0/2)
Total (%)	87 (42.9)	41 (20.2)	23 (11.3)	15 (7.4)	14 (6.9)	13 (6.4)	10 (4.9)	203	(90/113)

* M/F= Male/Female

**Table 2:** Frequencies of dermatophytosis in Guilan based on gender and age

**Age group (years)**	**Type of infection**															
	***T. pedis*** ** (F/M)**		***T. cruris*** ** (F/M)**		***T. corporis*** ** (F/M)**		***T. faciei*** ** (F/M)**		***T. manuum*** ** (F/M)**		***T. capitis*** ** (F/M)**		***T. unguium*** ** (F/M)**		**Total n %**	
0-10	0	1	4	2	1	1	1	1	1	0	3	3	0	0	18	8.9
11-20	1	1	3	8	1	3	0	6	1	1	1	2	0	0	28	13.8
21-30	8	2	14	16	2	3	1	0	1	2	0	0	1	2	52	25.5
31-40	7	3	10	6	4	0	2	0	0	1	0	0	1	2	36	17.7
41-50	7	2	10	3	1	0	1	0	3	1	1	0	0	3	32	15.8
51-60	1	1	3	5	5	0	0	0	0	0	0	0	2	0	17	8.4
60 <	5	2	2	1	1	1	2	0	1	1	0	0	0	4	20	9.9
Total	29	12	46	41	15	8	7	7	7	6	5	5	4	11	203	100

The zoophilic and geophilic agents were *T. interdigitale* (22.6%), *Microsporum canis* (4.9%), *T. verrucosum* (2.5%), and *M. gypseum* (1%). Except for *T. tonsurans* and *T. verrucosum*, the isolation rates of all the remaining species were higher in females. The frequencies of the dermatophyte isolates with regards to gender and different anatomic sites are summarized in Table 1. Four isolates of the study were submitted to the Centraalbureau voor Schimmelcultures Fungal Biodiversity center* (Utrecht, the Netherlands)* and accessioned as *T. verrucosum* CBS 130944 to CBS 130947.

## Discussion

Unlike the vast majority of reports from Iran and around the world [12-17, 20-21] and in consonance with a handful of other studies [7, 22-24], the incidence of dermatophytosis in our study was remarkably higher in females than males. This observation was not completely unexpected because it is known that the women living in the coastal regions of the Caspian Sea are engaged in outdoor activities such as animal husbandry, tea cultivation, and paddy field activities. Additionally, the use of traditional costumes, which cause excessive sweating, is popular among women living in Guilan. All these factors can account for the higher frequency of dermatophytosis in the studied women than in men.

In agreement with two previous reports from Iran [14, 17], involvement of the groins and the surrounding areas was the main clinical form among the studied patients, followed by tinea pedis and tinea corporis. Despite the fact that tinea cruris is normally far more common in men [1-3], in our survey, its infection rate was higher in women than men. 

In line with the studies performed in Tehran [11], Ahvaz [14, 25], and Qazvin [17], the infection occurred more frequently in the age group of 21-30 years, and contrary to the prior investigations from Iran [11-15, 17], our results showed a shift from the domination of *E. floccosum* towards *T. rubrum* in the casual agents of tinea cruris. Tinea pedis ranked second in frequency and accounted for almost a fifth (20.2%) of all the cases. Unlike the current prevailing trend, in which *T. rubrum* is the main species implicated in foot infection all over the world [2, 5], *T. interdigitale* was the main agent followed by *T. rubrum* and *E. floccosum *in Guilan. This finding was similar to some reports from Iran and abroad [12, 13, 26]. 

Ringworm of the foot usually affects men within the age group of 20-40 years [26], and in the present study, the infection mostly occurred in this age group; however, in terms of gender distribution, its prevalence rate was significantly higher in women due to the aforementioned reasons. Ringworm of the body had the third highest rate of occurrence (11.3% of all infections), and like the study of Vena et al. [27], it mostly affected women. 

In prior reports from different localities of Iran, the dermatophyte species like* E. floccosum* [13], *T. verrucosum *[15-17]*, T. interdigitale* [12, 14, 25], and* T. tonsurans* [28] were cited as the main agents of infection, while in the present study, *T. rubrum*, the main species involved in tinea corporis worldwide, was responsible for the most cases of infection. *Microsporum *spp., albeit less frequently, have been listed as the agents of tinea corporis in Iran [12-17, 28-30], amongst which *M. canis* and *M. gypseum* were the principal species, respectively. Moreover, these species were cited as the dominant agents of body infection in some European countries [20, 27]. However, no case of tinea corporis by such species was found in the Guilanian patients. Invasion of the nails (tinea unguium) ranked fourth in terms of frequency and constituted 7.4% of all the infections in our study. 

Generally, dermatophytic nail infections are more common in male and elderly people due to the higher possibility of nail trauma, slower rate of nail growth, and higher prevalence of tinea pedis in this population group [31]. Moreover, the infection is much more likely to be due to *Trichophyton* spp., and onychomycosis by *Microsporum* spp. is uncommon [32]. Congruent with these facts and similar to the study of Aghamirian and Ghiasian [17], in our study, nail involvement mostly occurred in men, and exclusively, the patients aged over 20 years. Likewise, *T. interdigitale* and *T. rubrum* were the predominant agents of onychomycosis in Guilan, and only one case of nail infection by *M. canis* was detected. 

Based on the recent molecular screenings, *T. interdigitale* is currently the main species causing nail infection in Iran [11, 12, 25], and in line with this finding, *T. interdigitale* was the predominant agent of tinea unguium in Guilan. Infection of the non-bearded areas of the face manifested with the same ratio in both genders and more frequently in the age group of 11-20 years. Generally, tinea faciei is a rare dermatophyte infection that is known to be more common among children and women and those in close contact with pets [33, 34]. Nevertheless, pets are less common among Iranians and the potential sources of infection are either infected individuals or domesticated animals such as dogs and livestock [12]. Congruent with this fact, the human-adapted species (e.g., *T. tonsurans*) was the principle agent of tinea faciei in Guilan, such that 6 out of the 11 *T. tonsurans* isolates were implicated in this form of tinea. In concurrence with the prevalence of infection in Khuzestan, Southwestern Iran [25], a significant rate of tinea faciei (6.9%) was observed in Guilan. This issue can be attributed to the propensity of this type of dermatophytosis for sultry climates [34], which is similar to that observed in Guilan and Khuzestan provinces. In earlier reports from Iran, the frequency of tinea manuum varied from 4.2% to 17.1% [12, 13], whereas in the current study, it accounted for 6.4% of all cases of dermatophytosis. Tinea manuum is an infection known to be most often caused by anthropophilic species [3]. In agreement with this fact and some reports from Iran and abroad [7, 13, 20], *T. rubrum*, *T. violaceum*, and *E. floccosum* were the predominant agents of tinea manuum in Guilan, respectively.

Until recently, ringworm of the scalp and hair was the foremost tinea in some parts of Iran [15, 16], but it had the lowest prevalence rate among our patients and predominantly occurred in preadolescent and adolescent samples. This finding was in congruence with the recent observations showing a gradual decline in the frequency of tinea capitis in Iran [11, 12, 17, 25]. However, tinea capitis remains an unresolved problem in Southwestern Iran [25]. In different investigations, *T. violaceum* [13], *T. tonsurans* [28], *M. canis* [12, 14], *T. verrucosum* [14, 15, 17], and *T. schoenleinii* [16] were enumerated as the main causes of tinea capitis in different areas of Iran. In the current survey, the etiologic agents of tinea capitis were mostly limited to zoophilic species of *M. canis* and *T. verrucosum* (n=7) and *T. tonsuransi*, which reinforce this theory that contact with cats, dogs, and domestic animals is the main mode of tinea capitis acquisition in Iran [25]. No case of infection by *T. schoenleinii* (favus) was encountered among our patients in Guilan. The progressive disappearance of this species in Iran and other parts of the world has been reported recently [12, 21, 25].

As concerns the causal agents*, the spectrum of pathogenic dermatophytes in Guilan was limited to eight species. *While an apparent reduction in the isolation rate of species like *M. canis*, *T. violaceum*, *T. verrucosum*, and *M. gypseum* was observed, the infections caused by *T. rubrum* have increased, and for the first time in our experience, the isolation rate of *this species *from patients with dermatophytosis was higher than that of other species in an area of Iran. T. rubrum* is known to constitute almost 70% of all dermatophytosis cases worldwide and infections by this human-adapted species* are difficult to treat and often associated with recurrence [35, 36],* which is of public health concern. This species is not nativeto Iran, but its ever-growing etiological role in dermatophytosis has recently become evident in Tehran and south of Iran [*11*, *12*, *18*]. *As previous investigations have stated [11, 12, 25], part of the reduction in the number of *T. verrucosum* isolates in Guilan can be attributed to the recent changes in the dermatophyte species [36], that is, some of the isolates that were morphologically identified as *T. verrucosum* (2 out of 7 isolates in this study) were actually *T. interdigitale* in ITS-RFLP analysis. Moreover, recent improvements in the breeding conditions of cattle, as the main reservoir for *T. verrucosum* infections, led to a decrease in the incidence of dermatophytosis by this zoophilic species [21].

## Conclusion

In conclusion, findings of this survey have proven that the distribution patterns of dermatophytes are notably variable in different parts of Iran. While an obvious decline in the rate of infection with anthropophilic species was observed in south of Iran, the emergence of *T. rubrum* as the main causative agent of dermatophytosis in Guilan, North of Iran, was remarkable. The study provided useful epidemiological data to establish therapeutic and preventive strategies for dermatophytosis in Guilan. To comprehensively monitor the epidemiological trends of dermatophytosis in any part of the country, further periodical and molecular-based investigations are necessary.
